# B‐cell capacity for differentiation changes with age

**DOI:** 10.1111/acel.13341

**Published:** 2021-03-12

**Authors:** Xuanxiao Xie, Jennifer Shrimpton, Gina M. Doody, Philip G. Conaghan, Frederique Ponchel

**Affiliations:** ^1^ Leeds Institute of Rheumatic and Musculoskeletal Medicine and NIHR Leeds Biomedical Research Centre University of Leeds Leeds UK; ^2^ Division of Haematology and Immunology Leeds Institute of Medical Research University of Leeds Leeds UK

**Keywords:** ageing, B‐cell differentiation, T‐cell dependent, T‐cell independent

## Abstract

**Background:**

Age‐related immune deficiencies are thought to be responsible for increased susceptibility to infection in older adults, with alterations in lymphocyte populations becoming more prevalent over time. The loss of humoral immunity in ageing was attributed to the diminished numbers of B cells and the reduced ability to generate immunoglobulin.

**Aims:**

To compare the intrinsic B‐cell capacity for differentiation into mature plasma cells (PCs), between young and old donors, using in vitro assays, providing either effective T‐cell help or activation via TLR engagement.

**Methods:**

B cells were isolated from healthy individuals, in younger (30–38 years) and older (60–64 years) donors. An in vitro model system of B‐cell differentiation was used, analysing 5 differentiation markers by flow cytometry, under T‐dependent (TD: CD40/BCR stimulation) or T‐independent (TI: TLR7/BCR activation) conditions. Antibody secretion was measured by ELISA and gene expression using qPCR.

**Results:**

TI and TD differentiation resulted in effective proliferation of B cells followed by their differentiation into PC. B‐cell‐executed TI differentiation was faster, all differentiation marker and genes being expressed earlier than under TD differentiation (day 6), although generating less viable cells and lower antibody levels (day 13). Age‐related differences in B‐cell capacity for differentiation were minimal in TD differentiation. In contrast, in TI differentiation age significantly affected proliferation, viability, differentiation, antibody secretion and gene expression, older donors being more efficient.

**Conclusion:**

Altogether, B‐cell differentiation into PC appeared similar between age groups when provided with T‐cell help, in contrast to TI differentiation, where multiple age‐related changes suggest better capacities in older donors. These new findings may help explain the emergence of autoantibodies in ageing.

## Graphical abstract

B‐cell differentiation into plasma cell appeared similar between age groups when provided with efficient T‐cell help. In contrast, in TI differentiation age significantly affected B‐cell proliferation, viability, depth of differentiation, antibody secretion and gene expression, suggesting better capacities in older donors.

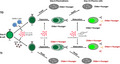

## 1 INTRODUCTION

There is no doubt that ageing is associated with multiple changes in different components of the immune system. The gradual deterioration of the immune system, often referred to as immunosenescence, affects the adaptive arm more than the innate one in humans (and rodents) (Han et al., 2003; Pangrazzi & Weinberger, 2020). In parallel, a state of chronic, low level inflammation (inflammageing) is observed in the elderly (Franceschi et al., 2000; Montecino‐Rodriguez et al., 2013). Primary dysfunctions in human T and B cells contribute to these age‐related aberrations, in addition to the relative loss of cells (Aspinall & Andrew, 2000; Aw et al., 2007; Fali et al., 2018; Goronzy et al., 2015; McElhaney et al., 2020; Quinn et al., 2018; Sansoni et al., 1993; Wagner et al., 2018). There is a general decline of T‐cell functions, exemplified by weaker activation of T cells resulting in poor proliferative capacity (Salam et al., 2013; Wagner et al., 2018). Effector functions of CD4^+^ T cells including antigen recognition (Goronzy et al., 2015) and killing capacity of CD8 T cells are reduced (McElhaney et al., 2020; Quinn et al., 2018) and thought to be responsible for an increased susceptibility to infection in older adults (Aw et al., 2007). Additionally, it is also well documented that human (and mice) T‐cell lymphopoiesis is reduced with ageing (Aspinall & Andrew, 2000; Fali et al., 2018; Sun et al., 2012), while this is less clear for human B cells post‐adulthood (Pang et al., 2011; Rundberg Nilsson et al., 2016) contrary to mice/rabbit that show a clear decline (Kennedy & Knight, 2017; Riley, 2013; Riley et al., 2017). Similarly, further to the total number of peripheral B cells being affected by ageing, antigen‐specific and polyclonal responses are reduced, as well as vaccine responses, with change in repertoire and telomere length (Bulati et al., 2011; Cancro et al., 2009; Crooke et al., 2019; Guerrettaz et al., 2008; Lin et al., 2016; Martin et al., 2015). Furthermore, studies suggested that ageing affects B‐cell selection resulting in higher frequencies of autoreactive cells being selected, which will directly influence autoantibody (auto‐Ab) production (Dunn‐Walters, 2016; Johnson & Cambier, 2004). Total levels of immunoglobulins (Igs) are slightly increased with age, however with differences in IgG and IgA levels going up while levels of IgM are reduced (Listi et al., 2006). The balance between effective response and tolerance is therefore compromised with age. This results in increased susceptibility to infection, chronic inflammatory disorders, frailty and increased risk of cancer development and autoimmunity. The increased capacity of B cell to generate auto‐Abs in the absence of suitable T‐cell help therefore remains to be fully explained.

In vivo, the B‐cell response to an antigenic challenge includes two waves of differentiation. An early response generates proliferation and the differentiation of memory B cells that produce germline‐encoded or sparsely mutated antibodies, as well as early plasmablasts (EPBs) that are short‐lived (Sabouri et al., 2016). Previous reports have shown that total CD19^+^ B cells from elderly donors (>65 years) are less able to differentiate after stimulation in vitro with polyclonal activators (CpG or SAC + IL‐2) that generate short‐lived plasmablasts in the absence of T‐cell help (Frasca et al., 2016; Shi et al., 2005). In a second phase, B cells undergo further expansion and affinity maturation within a germinal centre prior to terminal differentiation into PC, but are dependent on survival factors to become long‐lived PCs. In vitro assays were designed to study B‐cell differentiation into PC both quantitatively and qualitatively (Cocco et al., 2012; Shrimpton et al., 2020). These assays can use a T‐cell‐dependent (TD) or T‐cell‐independent (TI) stimuli to activate the B cells and thereafter push them towards differentiation into EPB, then a LPB and full PC status over 13 days. These in vitro assays allow to compensate for any age‐related factor that could affect the outcome, by providing controlled signals and environment to drive the generation of antibody‐secreting PC. Furthermore, both assays generate mature PC, with a phenotype and gene expression profile similar to ex vivo bone marrow‐purified PC (Cocco et al., 2012).

In this study, we aimed to gain insight into the intrinsic capacity of B cell to differentiate towards a full PC phenotype, comparing young and old healthy donors. Changes in the characteristics of the differentiating populations were quantified using these in vitro models of B‐cell differentiation, where T‐cell help is mimicked by a cell line expressing CD40L and TI stimulation is achieved using a TLR7 agonist, in combination with BCR stimulation (Cocco et al., 2012; Shrimpton et al., 2020). Generated PC numbers, their phenotype, secretary capability and gene expression profiles were compared.

## 2 METHODS

### 2.1 Blood samples

Ethical approval for this study was granted by the Leeds East Yorkshire Research Ethics Committee (reference: 07/Q1206/47). 15 healthy adult donors were included, divided into two groups depending on age: younger group including donors (age 30–38 years) and the older group including donors (age 60–64 years). Individual donor details are provided in Table S1. Peripheral blood was obtained from healthy donors after informed consent.

### 2.2 In vitro B‐cell differentiation assays

Mononuclear cells were isolated from EDTA blood, by density centrifugation followed by negative selection with a human B‐cell isolation kit (Miltenyi Biotec). Isolated B cells were cultured in 24‐well plates at 2.5 × 10^5^ live cells/ml in complete IMDM containing 10% heat‐inactivated foetal bovine serum (HIFBS; Invitrogen), non‐essential amino acids (Sigma) and lipid mixture (Sigma). B‐cell differentiation was induced as previously described (Cocco et al., 2012; Shrimpton et al., 2020), for both TI and TD stimuli. B cells were activated by the addition of F(ab′)_2_ goat anti‐human IgM and IgG (10 μg/ml), in the presence of h‐IL‐2 (20 U/ml) and h‐IL‐21 (50 ng/ml). B cells were co‐stimulated by gamma‐irradiated CD40L‐expressing L‐cells (1 × 10^6^/plate, seeded 1 day in advance at 1 × 10^6^ cells/well) to mimic TD help. Alternatively, B cells were co‐stimulated with the TLR 7/8 agonist R848 (InvivoGen, 1 µg/ml) to generate a TI signal. Subsequently, cells were collected, spun and re‐suspended in fresh complete medium (as above) with IL‐2 and IL‐21 at day 3 and grown until day 6. Gamma‐irradiated HS‐5 cells (ATCC^®^ CRL­11882™) were seeded (5 × 10^4^ cells/ml) in 96‐well round‐bottom plates 1 day in advance. Activated B cell at day 6 was harvested as above and seeded at 1 × 10^6^ live cell/mL for long‐term differentiation into plasma cells with the support of HS‐5 cells in complete medium in the presence of IL‐6 (10 ng/ml), IL‐21 (50 ng/ml) and interferon‐α (100 U/ml) up to day 13. 50% fresh complete medium (with cytokines) was replaced at day 10.

Cells were counted at each step of the culture, and all assays were normalized for cell numbers at days 6 and 13. Final results are presented to reflect output generated from 1 million live B cells at day 0 for all donors with different proportions of dead cells. Note that between day 6 and day 13, a large proportion of dead cells have transformed into apoptotic bodies and small debris and as such are no longer counted as a ‘cellular’ event.

### 2.3 Flow cytometric analysis

Cells were analysed using seven‐colour direct immunofluorescence staining on a CytoFLEX LX Flow Cytometer (Beckman Coulter). Abs used were CD20‐AF700 (clone 2H7), CD27‐PE (M‐T271), CD38 PE‐Cy7 (HB7), CD24‐FITC (ML5) and IgD‐V500 (IA6‐2), all from BD Biosciences with CD138‐VioBlue (44F9) (Miltenyi). In a second panel, CD20, CD27, CD38, CD138 and IgG‐V500 (G18‐145); IgM‐APC (G20‐127) (both BD Biosciences); and IgA‐FITC (IS11‐8E10) (Miltenyi) were also tested on circulating B cells (day 0 only). Cell populations were gated on FSC and SSC plot to remove small debris events (including apoptotic bodies), while a total event gate recorded viable and dead cells (gated using the 7AAD dye 50 µg/ml [BD Biosciences]). Absolute cell counts were calculated using CountBright beads (25,000 beads/panel; Invitrogen). Analysis was performed with CytExpert software, version 2.3 (Beckman Coulter).

A classic gating strategy for positive/negative live cell events was used (see profile in Figure S1) to evaluate changes in marker positivity at days 0, 6 and 13 and reported as % of total live cells. A gating strategy to define circulating naïve/memory from B‐reg and EPB, and expression of surface Ig is described in Figure S2.

### 2.4 viSNE and SPADE analysis

viSNE (visualization of t‐distributed stochastic neighbourhood embedding; https://www.cytobank.org/; Amir el al., 2013) maps were generated to visualize expression levels of CD24, CD27, CD38, CD138, and IgD. Analysis was performed using the default t‐SNE parameters. Clustering analysis of the viSNE output was then performed using SPADE (Spanning‐tree Progression Analysis of Density‐normalized Events; https://www.cytobank.org/). Clusters of nodes were then labelled manually according to cell phenotype.

### 2.5 Total IgM/A/G secretion

Supernatants from B‐cell differentiation assays were collected at day 6 and day 13 for total IgM‐, IgA‐ and IgG‐level measurements using commercial enzyme‐linked immunosorbent assay (ELISA) according to the manufacturer's instruction (Bethyl Laboratories).

### 2.6 Gene expression

Total RNA was extracted using the Direct‐Zol™ RNA MiniPrep (Zymo Research). Complementary DNA (cDNA) was reverse‐transcribed from total RNA by High‐Capacity cDNA Reverse Transcription Kit (Applied Biosystems). Gene expression was quantified using TaqMan^®^ assays for BCL‐xL (*BCL2L1*; Hs00236329_m1), *BCL2* (Hs00608023_m1), *BAX* (Hs00180269_m1), *BAD* (Hs00188930_m1), *MCL1* (Hs01050896_m1), *XBP1* (Hs00231936_m1), *AICDA* (Hs00757808_m1), BLIMP‐1 (*PRDM1*; Hs00153357_m1) and AIOLOS (*IKZF3*; Hs00232635_m1) according to the manufacturer's instructions (Applied Biosystems) and normalized using *UBC* gene (Hs00824723_m1) as housekeeping.

### 2.7 Data analysis

All statistical analysis was performed using SPSS Statistics 26 (IBM) and GraphPad Prism™ v8 software. ANOVA was used (with Dunn's well correction if applicable), and differences were tested individually by the Mann–Whitney *U* test (MWU). Correlations were calculated by the Pearson and Spearman coefficient. *p*‐value <0.05 was considered significant.

## 3 RESULTS

### 3.1 Comparison of TD and TI differentiation pathways

**FIGURE 1:**
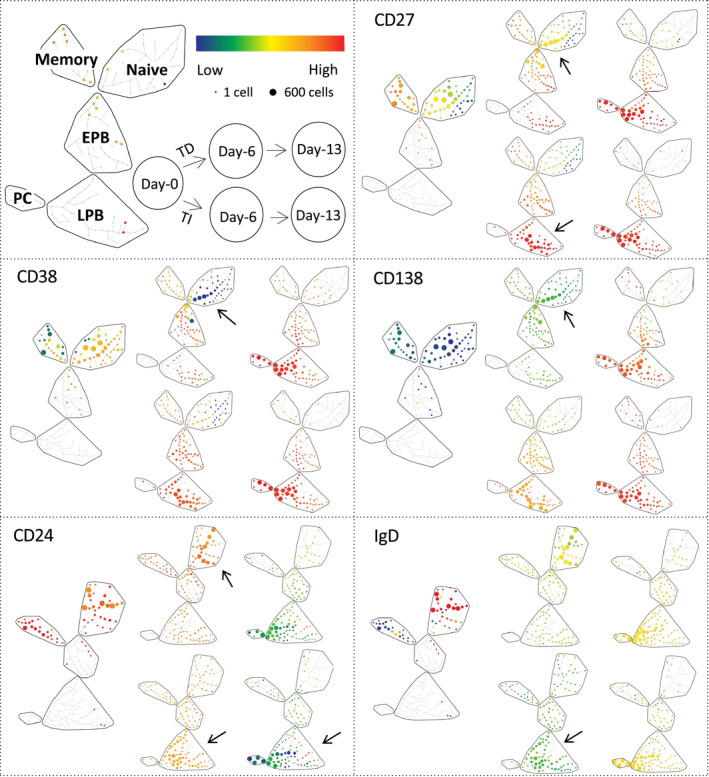
B‐cell changes in marker expression levels during differentiation. *Top left*: SPADE trees of cellular hierarchy with clusters of cells (nodes) with a similar profile being grouped in subsets (region: naïve, memory, EPB, early plasmablasts; LPB, late plasmablasts; PC, plasma cells) based on manual annotation of the phenotype. Organization of data displayed for TD/TI differentiation at 3 time points (days 0, 6 and 13) and colour‐coded scale for low (blue) to high (red) levels of expression. *Top right and bottom 4 panels*: SPADE trees of cellular hierarchy for 5 markers expressed on B cells in a representative donor (age 36). Arrows highlight visible differences. Note that regulatory B cells are not indicated on the SPADE representation (usually part of the EPB subset) due to their small number and only possible identification at day 0. Also, note the move from naïve and memory subsets to EPB and LPB at day 6 in TD while mainly from the memory to EPB subset in TI. Both assays showed very similar cell distributions in LPB and PC by day 13

We first directly compared outcomes of the differentiation models (TD or TI). Both differentiation assays were performed in 15 individuals. Throughout both assays, cells moved from one phenotypic subset to another (Figure 1, representative example from 1 donor, age 36), in TD, mainly memory cells progressed to EPB at day 6, while in TI, both naïve and memory cells had progressed to EPB and LPB. At day 13, the end result of the differentiation assays was similar, with cells accumulating in the LPB and PC categories. Surface marker expression followed expected patterns of change with progressively stronger expression of CD27 and CD38 accompanied by a loss of CD24 and IgD. The overall outcome of both pathways was similar, with B cells generating intermediate PB (CD38^+ ^CD138^−^), followed by the acquisition of CD138 as the cells reach the final plasma cell stage.

**FIGURE 2:**
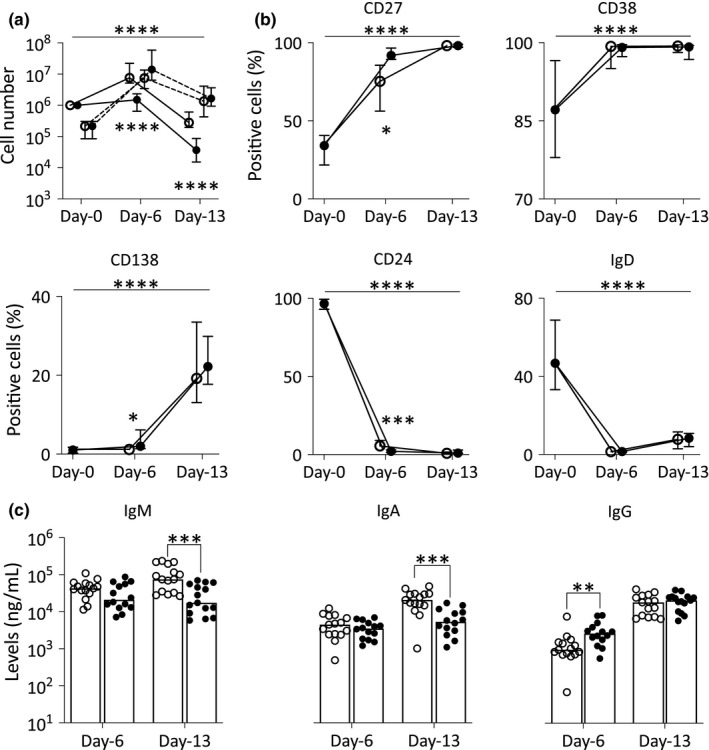
B‐cell changes in marker expression (% of positive cells) during differentiation. (a) Number of live (plain line) and dead (dashed line) B cells normalized (using counting beads) compared with day 0 (*n* = 15, median, IQR). (b) Positivity (% of positive live B cells) for each marker (median, IQR). (c) Total IgM, IgA and IgG levels measured by ELISA (*n* = 15) from supernatant collected at days 6 and 13. *p*‐Value for the overall results between two differentiation assays in (a) and (b) was calculated by ANOVA as indicated by the top bar. Comparison of the 2 assays at individual time points (all 3 panels) was tested by MWU, and significance is indicated by symbol next to each group (*n* = 10). ○, TD assay; ●, TI assay. *p*‐Values: *p < 0.050; **p = 0.009; ***p < 0.001; and ****p = 0.0001

Importantly, the number of live/dead cells generated over both assays was different (Figure 2a; top corner ANOVA, *n* = 15, *p* = 0.0001) showing highly significant difference at day 6 with a mean 8‐fold more live cells generated in TD but only 1.6‐fold in TI (*n* = 15, *p* = 0.0001). This was also observed at day 13 with 0.7‐fold in TD and 0.07‐fold in TI (*p* = 0.0001). In contrast, there were more dead cells generated at day 6 in TI (153‐fold increase) than in TD (40‐fold, *p* = 0.050).

Furthermore, differences in marker expression were visible between the two different assays, particularly at day 6 (Figure 1; arrows). In TI differentiation, B cells generated subpopulations that were spread across multiple nodes of significant size within the EPB subsets, LPB and PC segment of the SPADE tree. In contrast, in TD differentiation, B cells remained spread within only a few nodes away from the naïve and memory subsets. The levels of marker expression also differed in TI compared with TD at day 6. In further support to this observation, a small proportion of B cells under TI condition had already acquired extensive CD38 expression at an significantly increased frequency, paired with increased CD27 levels/frequency and higher expression/frequency for CD138, while decreased levels/frequency of CD24 and IgD were observed compared with TD (Figure 2b; ANOVA, *n* = 14, *p* < 0.050 and individual time point significance by MWU indicated in figure). At day 13, CD27, CD38 and CD138 marker expressions were high (Red levels), while for CD24 and IgD, the cells showed low levels of expression (Green/Yellow) in both differentiation assays. The distribution of the populations across the subset segments was relatively similar between both types of stimulation.

We also investigated the relative production of Ig isotypes in the context of TD versus TI differentiation, looking at secretion of IgM, IgA and IgG at days 6 and 13. For IgM and IgA, limited differences were observed at day 6, with lower levels detected in TD compared with TI differentiation at day 13 (Figure 2c; MWU, *n* = 15, both *p* < 0.010). In contrast, levels of IgG secreted were higher during TI differentiation at day 6 (*p* = 0.00), but similar at day 13.

We therefore observed differences in the final results of the differentiation assay with a clear higher live plasma cell number generated by TD compared with TI. A faster progression through differentiation was observed in the TI assay, with the presence of more cells in the EPB and LPB subsets (on SPADE tree, earlier gained in CD27^+^ and CD138^+^ cells, loss of CD24^+^ and IgM^+^ cells), associated with IgG secretion at day 6, which suggest that cells had already reached a more advanced stage of differentiation, but have not been able to survive the early phase of expansion affecting the number of viable cells detected at days 6 and 13. As such, a higher total number of B cells (live + dead, mean 154‐fold increase) were observed in TI at day 6 accounting for an earlier and more effective proliferation phase compared with TD (63‐fold increase). CD24 expression undergoes continuous expression fluctuations throughout the lifespan of B cells until CD24 is lost when B cells differentiate into antibody‐producing cells (Mensah et al., 2018). As such, the expression of CD24 reduces more profoundly in TI in all compartments of the SPADE tree from circulating and day 6 and day 13 B cell. To further support this hypothesis, we also observed faster progression to late differentiation stages in the TI assay as manifested by more pronounced gain or loss of all marker's expression at day 6, while being more similar at day 13.

We also compared male (*n* = 7) and females (*n* = 8) and observed no significant differences in circulating subset frequency, marker levels of expression, % of + cells, Ig production and gene expression between gender groups (Figures S3, S4 and S7), while cell counts showed a trend for higher live cell means during female B‐cell expansion in both TD and TI assays at day 6, as well as for the generation of live PC at day 13 (not significant).

### 3.2 Age‐related differences in circulating B cells

We then analysed circulating B‐cell phenotype comparing age groups (younger *n* = 8, and older donors, *n* = 7). Using a classic flow cytometry manual gating analysis, subsets defining naïve (CD24^+ ^CD27^− ^CD38^− ^CD138^− ^IgD^+^), memory (CD24^low ^CD27^+ ^CD38^low ^CD138^− ^IgD^−^), B‐reg (CD24^high ^CD27^− ^CD38^high ^CD138^− ^IgD^+^) or EPB (CD24^− ^CD27^+ ^CD38^+ ^CD138^low ^IgD^−^) were quantified (Figure S5). Despite not reaching significance in such a small group, trends for higher naïve, lower memory and similar EPB previously observed (Chong et al., 2005; Ponchel et al., 2015; Shi et al., 2005) were reproduced in this small group. We also observed a higher proportion of B‐reg in younger donors, which reflects other published data (Duggal et al., 2013).

**FIGURE 3:**
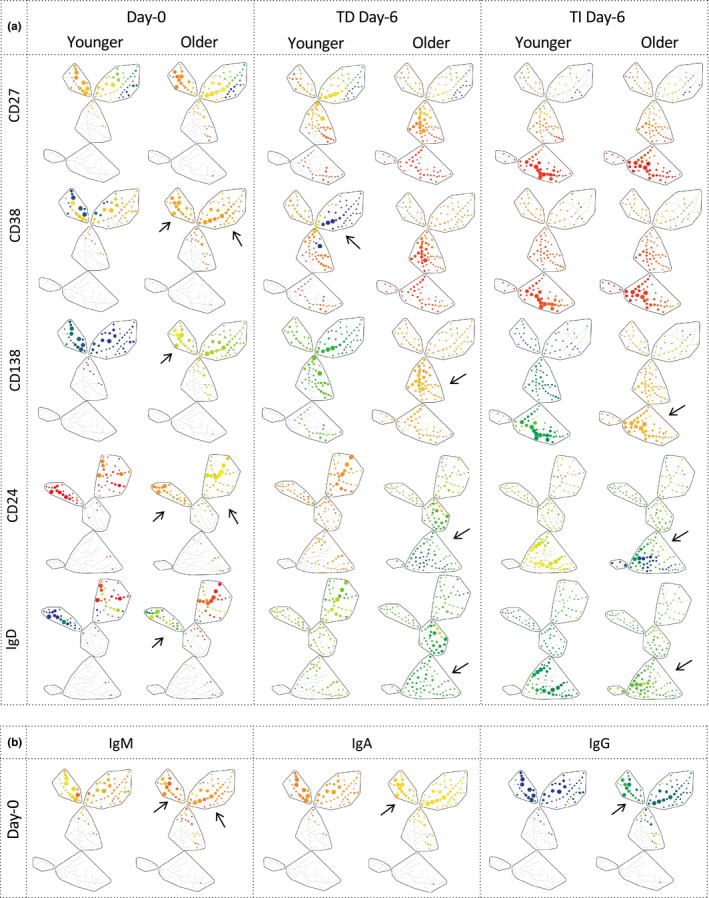
B‐cell changes in marker expression levels at day 0 and day 6 of differentiation in young and old subjects. SPADE trees of cellular hierarchy in a representative donor (age 36 and 61) for (a) 5 markers expressed on live B cells at days 0 and 6 of the assays (using the same display as in Figure 1) and (b) Ig‐isotype expression on live circulating B cells (day 0). Arrows highlight visible differences in levels of expression of markers. Note that the proportion of naïve and EPB cells is higher in the older donor at day 0 (as shown by larger nodes in these subsets). Also, note the move from naïve and memory subsets to EPB in older donor at day 6 in TD while mainly from the memory to EPB subset in the younger one. In contrast in TI, both donors showed very similar cell distribution in EPB and LPB by day 6

**FIGURE 4:**
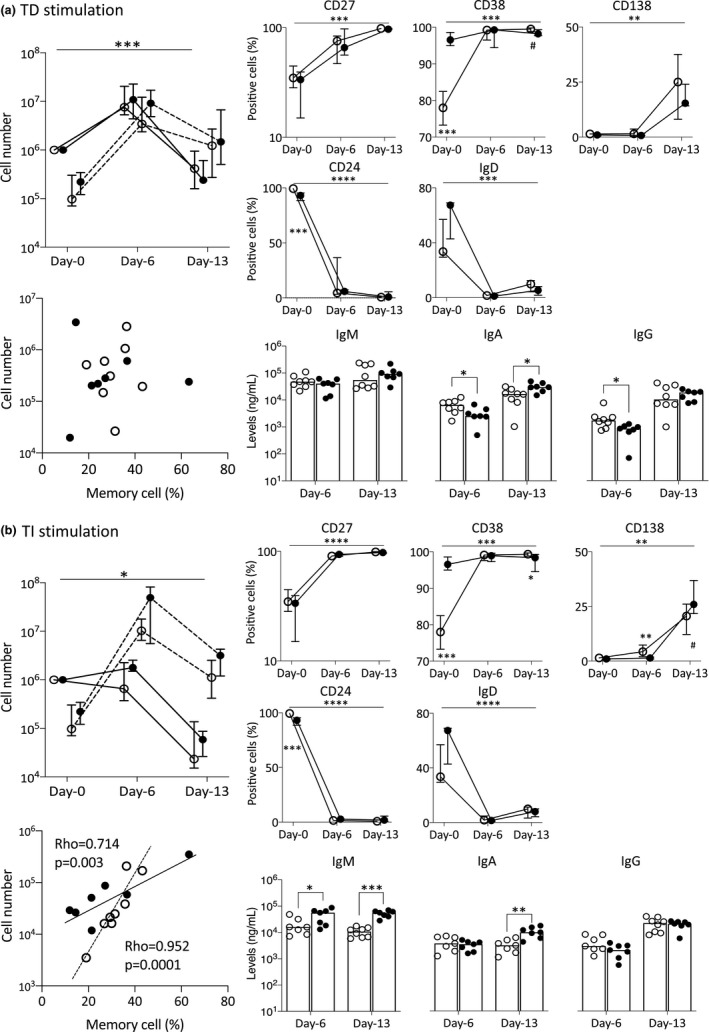
Changes in B‐cell differentiations between age groups during (a) TD and (b) TI stimulation conditions. *Top left*: Number of live (plain line) and dead (dashed line) B cells at different time points. *Bottom left*: Relationship between % memory cells in circulating B cells and number of PC generated at day 13. *p*‐Value and rho were calculated using the Pearson correlations. *Top right*: Positivity (% of live cells) for each marker (median; IQR). *Bottom right*: Total IgM, IgA and IgG levels measured from supernatant collected at days 6 and 13. *n* = 8 younger (1 donor missing CD24, IgD and IgM/A/G in TI) and *n* = 7 older. *p*‐Value for the overall assays between groups was calculated by ANOVA (top bar). For individual comparison between 2 groups at a time point, MWU test results are given next to the group denoting difference between old and young donors. ○, younger donors; ●, older donors. *p*‐Values: ^#^ *p* < 0.100; *p < 0.050; **p < 0.010; ***p < 0.001; and ****p = 0.0001

Using viSNE analysis and SPADE trees (Figure 3, day 0 panels a and b, representative example from 1 donor of each age group for both assays), higher levels of expression for CD38, CD138, IgD and IgM but lower CD24 and IgA were observed in older donors (arrows), also reflected in significant differences in percentages of CD38^+^ (higher) and CD24^+^ (lower) B cells (Figure 4, day 0 MWU, *n* = 8 for young and *n* = 7 for older groups, both *p* < 0.001). IgM^+^ cells also showed reduced frequency in older donors (*p* = 0.018, data not showed), however with higher levels of expression.

### 3.3 Age‐related differences in differentiation capacity

The expansion of viable B cells during TD differentiation was similar between younger and older donors (Figure 4a) with a mean 12‐fold increase for younger and 14‐fold increase for older donors. There was a non‐significant 1.5‐fold more dead cells at day 0 in older donors. At day 6, similar increases in dead cells were seen (not significant by 39‐fold in younger and 41‐fold in older), observed at day 13 (9‐fold in younger and 20‐fold in older). In contrast, during TI differentiation (Figure 4b) B cells showed more expansion at day 6 in older donors (mean 2‐fold increase) than in younger donors (1.3‐fold reduction, *p* = 0.189). The number of dead cells was also increased by 74‐fold in younger donors but by 198‐fold in older ones (*p* = 0.054) at day 6, suggesting more proliferation (i.e., live + dead) but less survival in older donors. In both assays, the number of live PC was reduced significantly by day 13 as expected, and there was no significant difference in live PC numbers at day 13; however, cells were 2‐fold more numerous in older donors in TI differentiation (mean 1 × 10^5^ live cells) than in younger ones (6 × 10^4,^) but more equal in TD differentiation (mean 7 × 10^5^ in older donors compared with 7 × 10^5^ in younger ones).

We cannot fully exclude that the initial number of memory B cells present in the blood can influence the outcome of the assays. There were less memory cells in older individuals, but this did not seem to affect the TD assay (Figure 4; bottom left corner), while it had a clear positive effect on the TI assay, with highly significant correlation between % of memory cells at day 0 and the number of PCs generated at day 13 in both younger (*ρ* = 0.952, *p* = 0.0001) and older donors (*ρ* = 0.714, *p* = 0.003).

During both assays, the expression of all markers followed the expected pattern. At day 13, very limited differences were observed (data shown in Figure S6). Focussing on day 6 and only on age‐related differences, CD27 mirrors the pattern of CD38 but with less intense expression (Figure 3, representative example from 1 donor of each age group for both assays) or frequency (Figure 4, % of positive cells). CD38 showed a faster increase in older donors in TD differentiation but similar expression in TI; however, this was not reflected in % of positive cells. CD138 increased more strongly both in TI and in TD in older donors, while positive cell frequencies were similar in TD but lower in TI for older donors (both day 6 and day 13). CD24 and IgD expressions were both lost quickly (almost no positive cells in either assays by day 6; no significant change), while at the levels of expression, both markers were lost more profoundly in older donors reaching darker green/blue levels but still orange/light green in younger ones.

After day 0, IgM secretion showed no difference between age groups during TD differentiation, while an increase in older donors was detected at both day 6 and day 13 in TI differentiation (Figure 4; MWU, *n* = 8 in younger and *n* = 7 in older group, *p* < 0.010). The levels of secreted IgA were higher at day 13 in older donors in both differentiation assays. Increased IgG secretion was observed at day 13 in both assays with no difference between younger and older donors.

### 3.4 Gene expression of survival and transcriptional regulators during differentiations

As we observed differences both in expansion and in survival, as well as depth of differentiation, 9 genes were chosen to indicate survival/cell death signals during differentiation: *BCL2*, *BAX*, *BAD*, *BCL2L1* (*coding for* BCL‐xL) and *MCL1*, as well as regulator of B‐cell maturation: *XBP‐1*, *PRDM1* (coding for BLIMP1 protein), *AICDA* and *IKZF3* (for Aiolos).

**FIGURE 5:**
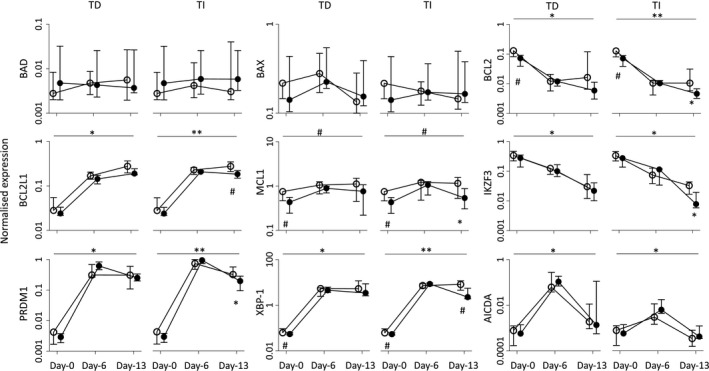
Expression profile of gene associated with differentiation and survival/apoptosis. Gene expression changes (median, IQR) during differentiation assays between age groups at different time points, quantified by TaqMan qPCR (*n* = 5 for each group). Overall changes between two groups were tested by ANOVA (top bar). Individual time point differences were tested by MWU. ○, younger donors; ●, older donors. *p*‐Values: ^#^ *p* < 0.100; *p < 0.050; **p < 0.010, ***p < 0.001; and ****p = 0.0001

At day 0, the B‐cell population from younger and older donors include different proportions of naïve, memory and other cell subsets (Figure S5). Gene expression profiles will be different in such subsets (Shen et al., 2004) and may therefore impact our ability to observe age‐associated changes. Using a small number of donors here (*n* = 5 for each group), age‐related difference for the 9 genes chosen was indeed limited, although we observed a small reduction in expression for *BCL2*, *MCL1* and *XBP‐1* in older donors (Figure 5; *n* = 5 for both age groups, MWU at day 0, all *p* < 0.100).

During both differentiation assays, levels of expression mostly followed expected patterns (Schmitt et al., 2002; Shapiro‐Shelef & Calame, 2005; Vikstrom et al., 2016) with a pronounced increase (both day 6 and day 13) for *BCL2L1*/BCL‐xL, *XBP‐1* and *PRDM1*/BLIMP1 or decrease for *BCL2* and *IKZF3*/Aiolos, while *AICDA* displayed only a transient increase. *BAD* and *BAX* expression remained unchanged (Figure 5, *n* = 5 per age group, ANOVA, top bars). Although *PRDM1*/BLIMP1 increased in response to both types of stimuli, the levels at day 13 dropped significantly in cells undergoing TI differentiation but less in TD.

Contrary to the phenotypic analysis, most significant differences between age groups were observed at day 13, at a time when most cells have reached the same PC stage, allowing age‐related difference to be observed. This was seen for *MCL1*, *XBP‐1* and *BCL2L1*/BCL‐xL, BCL2, *PDRM1*/BLIMP‐1 and *IKZF3*/Aiolos (Figure 5, *n* = 5 per group, MWU, all higher in younger donors, all *p* < 0.05) although with small differences. Most importantly, differences were observed only when induced by TI differentiation, while not observed under TD differentiation.

Of note, a gender analysis of the same data showed no significant difference in gene expression between males and females (Figure S7), with trends for lower *BCL2* and *MCL1* and *IKZF3*/Aiolos in females at day 0.

## 4 DISCUSSION

Altogether, our data showed that TD differentiation and TI differentiation of B cells follow similar patterns in terms of phenotypic change and gene expression, although it happened faster during TI, but generated more PC in TD. Age affected circulating B‐cell subsets as expected, and gene expression results showed change in the balance of survival factors (higher *BCL2* and *MCL1* in younger donors). B‐cell differentiation capacity appeared relatively similar between donors when using T‐cell help, while some changes in overall PB and PC phenotype were observed in older donors. In contrast, TI differentiation after co‐engagement of BCR and TLR was associated with age‐related changes in all parameters tested.

The blood composition of circulating B‐cell subsets has been shown to change with age affecting naïve and memory B cells, as well as B‐reg, but not the frequency of EPB (Chong et al., 2005; Duggal et al., 2013; Ponchel et al., 2015; Shi et al., 2005). Our data recapitulated these observations. Apoptosis is involved in several age‐related process including T‐cell immunosenescence (Ginaldi et al., 2004; Longo et al., 2005). Despite the possible differential gene expression contribution from B‐cell subsets (naïve/memory), age appears to affect anti‐apoptotic molecule expression *BCL2* and *MCL1* (but not *BCL‐xL*) but not the pro‐apoptotic factors (*BAX* and *BAD*). Older donors have more naïve cells but express less *BCL2*, which is further reduced during differentiation, suggesting a true age‐related change. *MCL1* expression remains lower in older individuals, which also suggests a true age‐related difference. MCL1 has multiple roles in T‐cell‐dependent B‐cell maturation (Peperzak et al., 2013; I. Vikstrom et al., 2010). As such, its expression appears to raise continuously in TD (notably in younger donors) but is reduced in TI (and in older donors). These suggest a particular age‐related change in MCL1 activity that needs further exploration. *BCL2L1* expression was shown to provide alternative B‐cell survival signals to PCs (Amanna et al., 2003). The lower expression of *BCL2L1* in older donors may therefore also contribute to the difference in PC numbers. Short‐lived PB at day 6 may have a better capacity for developing long‐lived PCs in aged donors particularly in TI, which was also observed in the bone marrow (Pritz et al., 2015). However, these changes are modest and may not be sufficient to explain the age‐related higher live cell numbers, suggesting that other ageing mechanisms may contribute to cell viability, as observed in lymphomas (Adams et al., 2018; Agarwal & Naresh, 2002).

Certain markers of B‐cell differentiation subsets were previously associated with age (CD38‐ and CD24‐positive cells) (Buffa et al., 2013; Chong et al., 2005). Our data support these findings but also add information about levels of expression showing age‐associated difference for all markers. These suggest that the function associated with such molecules may indeed alter the way B cells respond to stimuli and differentiate in ageing.

The in vitro assays used activate all B cells both from the naïve and from the memory subsets, then push their differentiation towards the EPB and then LPB and PC subsets. Analysing data with respect to age showed that in the presence of T‐cell help, older donors displayed a clear effect on the speed of progression from the naïve subset into EPB (larger nodes) and LPB (more nodes) but not from the memory subset (similar loss of sizeable nodes). This finding is clearly important but could only be detected using novel flow cytometry analytic techniques (not detectable with a classic % of positive cells). In TI, naïve and memory B‐cell subsets both engage in differentiation and reached LPB by day 6 and PC subsets by day 13. This may explain the higher cell numbers observed early in the TI response. However, only the memory compartment may mature effectively into PCs, explaining the relationship observed between % of memory cells at day 0. In contrast, in TD, naïve cells engage in maturation more slowly (particularly in younger donors), possibly leading to a better overall attainment of final PC number.

Despite the fact that these assays cannot fully reproduce microenvironmental factors in the bone marrow, a comparison of gene expression profiles between in vitro‐derived day 13 PC and ex vivo bone marrow PC confirmed a high degree of concordance and no change in the core gene expression programme (Care et al., 2016; Cocco et al., 2012; Stephenson et al., 2019). Transcription factors control B‐cell differentiation at different levels: late PC development (*PRDM1*/BLIMP‐1, *XBP‐1* [Shapiro‐Shelef et al., 2003; Turner et al., 1994]); endoplasmic reticulum adaptation to secrete Igs (*XBP‐1* and *IKZF3*/Aiolos [Iwakoshi et al., 2003; Schmitt et al., 2002]); and Ig‐class switching and hypermutation (*AICDA* [Di Noia & Neuberger, 2007]). *PRDM1* was more upregulated at day 6 in the aged donors (both assays), in line with an accelerated execution of the early differentiation. *XPB‐1* was upregulated, however, with limited difference between age groups, in line with the acquisition of the secretory capability at day 6 (Cenci & Sitia, 2007), and increased secretion at day 13, although additional age‐related changes in metabolic fitness (associated with mTOR) may also influence a B‐cell's ability to secrete antibodies (Stallone et al., 2019; Tellier & Nutt, 2019).

Altogether, the use of these assays allowed us to quantify the intrinsic capacity of B cells to differentiate into PC in the presence of T‐cell help directly compared with an alternative using TLR stimulation. Looking into the age‐related changes, our data showed that B cells have limited intrinsic quantitative difference in their capacity for differentiation in the presence of controlled T‐cell help. This is manifested by a similar capacity for proliferation at the early stage (day 6) (55‐fold increase (live + dead cells) in older donors compared with 51‐fold in younger ones), and resulting in similar number of PC at day 13. However, the depth of differentiation was more pronounced in older donors (higher expression of CD27, CD38 and CD138 in EPB and LPB and weaker expression of CD24 and IgD at day 6). In contrast, in TI differences were observed with increased early proliferation (200‐fold in older donors, compared with 73‐fold in younger ones), suggesting a better proliferative capacity of older B cells, associated with higher number of PC generated at day 13 (although not significant in this small group). Again, this was associated with a faster and more pronounced differentiation in older donors. In keeping with these observations, older donors produced antibody equally well to younger donors under TD differentiation (~1.3‐fold difference), but they appear to be more effective during TI, secreting more IgA and IgM (>3‐fold and >5‐fold, respectively).

In conclusion, the use of controlled in vitro differentiation assays combined with novel methods to analyse flow cytometry data allowed an evaluation of the impact of ageing on the generation of fully mature PC with or without T‐cell help. Our results suggest that B cells from older donors (who have no health issues) remain responsive to antigenic challenge in the context of in vitro provision of adequate T‐cell help/cytokines, while responding more efficiently than young donor B cells to co‐engagement of the BCR + TLR.

While we observed limited difference between gender groups, females appear to have better B‐cell proliferative/survival capacity than males, which may contribute to better responses to infection seen in older females compared with males (notably as observed in the COVID‐19 pandemic) (Gadi et al., 2020; Klein & Flanagan, 2016). Moreover, a particular subset of memory B cells (double negative IgD^− ^CD27^−^), which are thought to be responsible for the production of autoantibodies (Claes et al., 2016; Jacobi et al., 2008; Moura et al., 2017), is increased in elderlies (Frasca et al., 2019) (although no difference was observed in our group of donors), as well as in patients with autoimmunity. It will therefore be of interest in the future, to assess whether our in vitro differentiation models could be used to provide further understanding of the role of these cells in ageing and age‐related autoimmunity.

## CONFLICT OF INTEREST

No conflicts of interest, financial or otherwise, are declared by the authors.

## AUTHOR CONTRIBUTIONS

XX designed the study, performed all experiment, analysed data and drafted the manuscript. JS performed some experiment. GD analysed data and drafted the manuscript. PG reviewed the manuscript. FP designed the study, analysed data and drafted the manuscript.

## Supporting information

Supplementary Material
