# Supplementary material for: B‐cell capacity for differentiation changes with age
Source: Aging Cell. 2021 Mar 12;20(4):e13341. doi: 10.1111/acel.13341 (PMC8045946; doi:10.1111/acel.13341)
Supplement: Supplementary file 1 — Supplementary Material [file ACEL-20-e13341-s001.docx]

Supplementary figures

**Title: B-cell capacity for differentiation changes with age**

**Authors:** Xuanxiao Xie^1^, Jennifer Shrimpton^2^, Gina M. Doody^2^, Philip G. Conaghan^1^, Frederique Ponchel^1^

Xuanxiao Xie <umxxi@leeds.ac.uk>;

Jennifer Shrimpton <jennifer.k.shrimpton@gmail.com>

Gina M. Doody <G.M.Doody@leeds.ac.uk>;

Philip G. Conaghan <P.Conaghan@leeds.ac.uk>;

Frederique Ponchel <F.Ponchel@leeds.ac.uk>:

**Address**

1. Leeds Institute of Rheumatic and Musculoskeletal Medicine and NIHR Leeds Biomedical Research Centre, University of Leeds, UK

2. Division of Haematology and Immunology, Leeds Institute of Medical Research, University of Leeds, UK

**Corresponding Author**

Dr. Frederique Ponchel,

Leeds Institute of Rheumatic and Musculoskeletal Medicine

St James Hospital, CSB, room 5.25

Leeds

LS9 7TF,

UK

Email: F.Ponchel@leeds.ac.uk

**Keywords:** Aging; B-cell differentiation, T-cell dependent, T-cell independent

***Table SUP-1:* Donor information.**

| **Groups** | **Participates** | **Age** | **Gender** |
| --- | --- | --- | --- |
| Younger | #1 | 36 | M |
|  | #2 | 34 | M |
|  | #3 | 32 | F |
|  | #4 | 32 | F |
|  | #5 | 35 | F |
|  | #6 | 33 | M |
|  | #7 | 30 | F |
|  | #8 | 38 | M |
| Older | #9 | 60 | M |
|  | #10 | 61 | F |
|  | #11 | 64 | F |
|  | #12 | 62 | F |
|  | #13 | 62 | M |
|  | #14 | 60 | M |
|  | #15 | 64 | F |


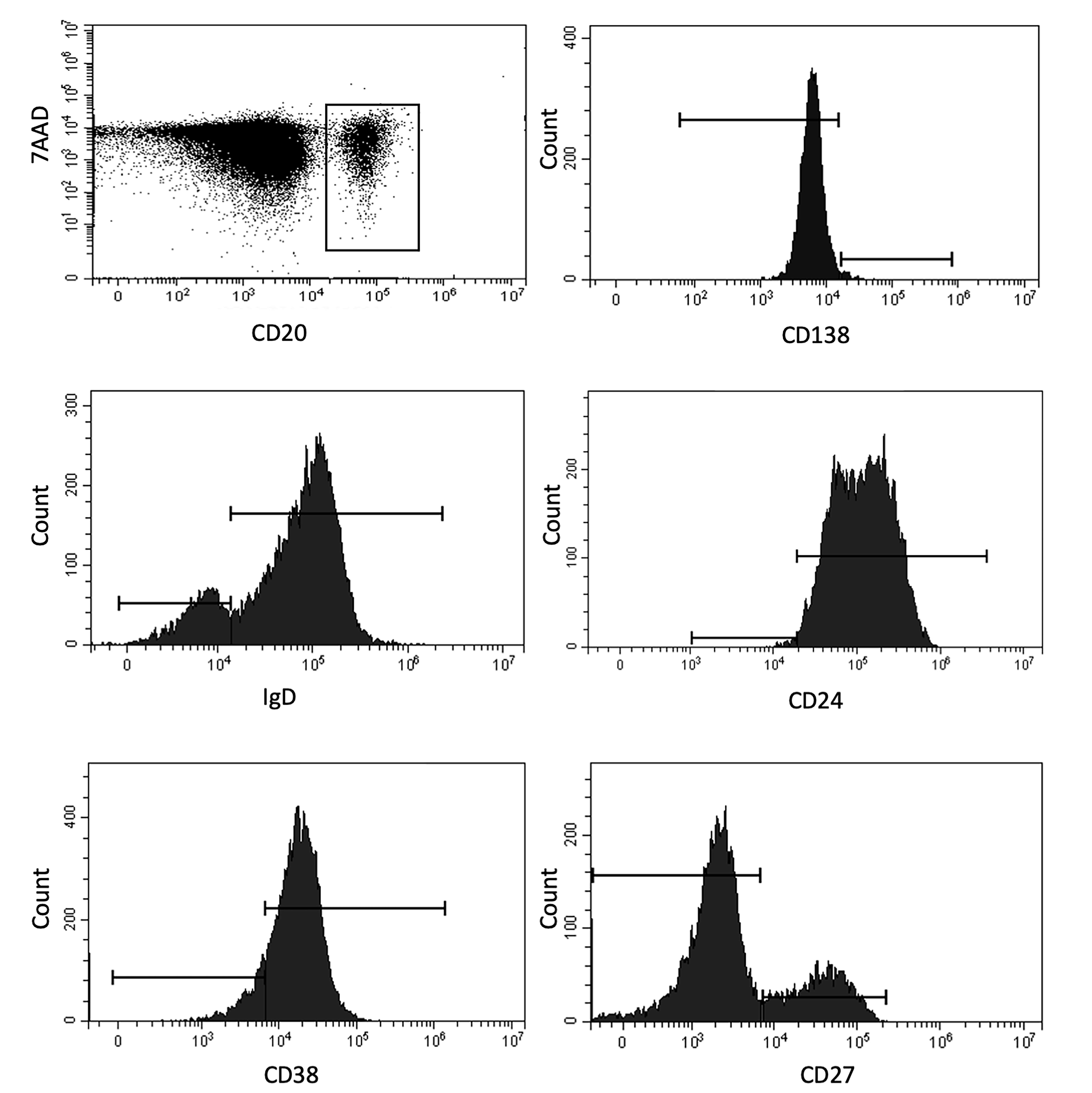


**Figure SUP-1 Gating strategy:** Circulating B-cells from a representative donor at day-0, were stained with antibodies against CD20, CD24, CD27, CD38, CD138, IgD and the live/dead cell dye 7AAD, and analysed by flow cytometry. Dead B-cells were first excluded (square), based on CD20^+^ expression and positivity for 7AAD. Histograms for each marker were then used to quantify positive or negative live B-cells.


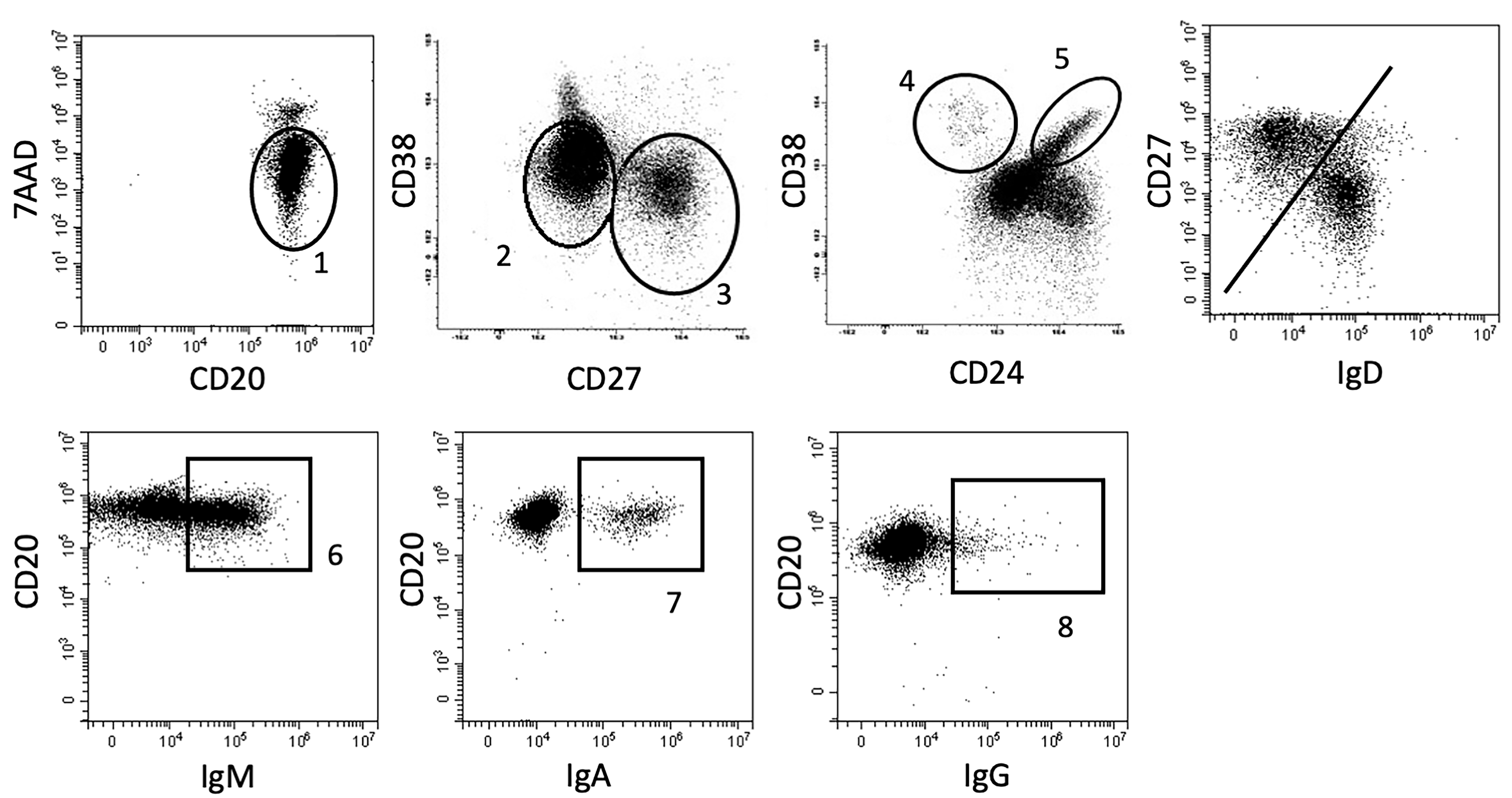


***Figure SUP-2:* Flow cytometry gating strategy for B-cell subsets frequency analysis at day-0.**

B-cell were stained with antibodies against CD20, CD24, CD27, CD38, CD138, IgD and 7AAD in tube-1 and anti CD20, IgM, IgA, IgG and 7AAD in tube-2.

Tube-1: Live cells (circle-1) were first gated by the exclusion of 7AAD^+^ cells in a dual plot with anti-CD20. Around 10% of dead cells were detected after purification on magnetic beads at day-0. An anti CD27 and CD38 dual pot was used to gate naïve (circle-2 CD20^+^CD27^-^CD38^+^), memory (circle-3 CD20^+^CD27^+^CD38^+^) cells. Anti CD24 and CD38 plot was used to gate EPB (circle-4 CD20^+^CD38^high^CD24^low^) and regulatory B-reg (circle-5 CD20^+^CD38highCD24high) subsets. IgD staining was positive on naïve B-cells (CD27^-^/CD38^+^) (as separated by the black line) and negative on most of the memory B-cells (CD27^+^/CD38^+^).

Tube-2: CD20+ live cells were gated as in tube-1 and ploted against anti-Ig-isotype. A large fraction of cells were IgM^+^ (square-6), and smaller ones were detected for IgA^+^ (square-7) and IgG+ (square-8) cells.


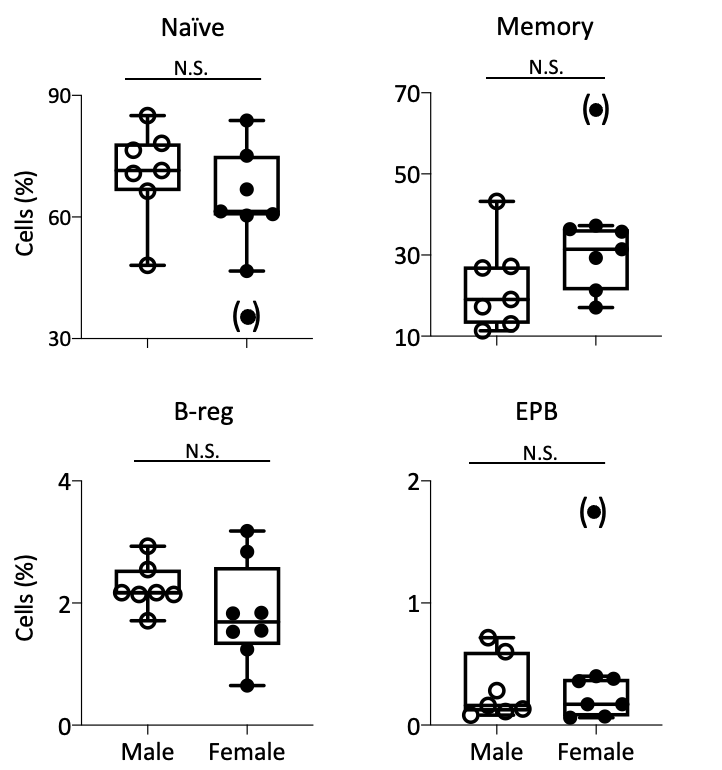


***Figure SUP-3:* Frequency of circulating B-cells subsets in the two gender groups***.* Male (n=7) and female group (n=8); 4 subsets were quantify using classic flow cytometry: naïve (CD24^+^CD27^-^CD38^-^CD138^-^IgD^+^), memory (CD24^low^CD27^+^ CD38^low^CD138^-^IgD^-^), regulatory B cells B-reg (CD24^high^CD27^-^CD38^high^CD138^-^IgD^+^) and early plasmablasts EPB (CD24^-^CD27^+^CD38^+^ CD138^low^IgD^-^). Outliners with brackets. *P-*value calculated by MWU. N.S. not significant.


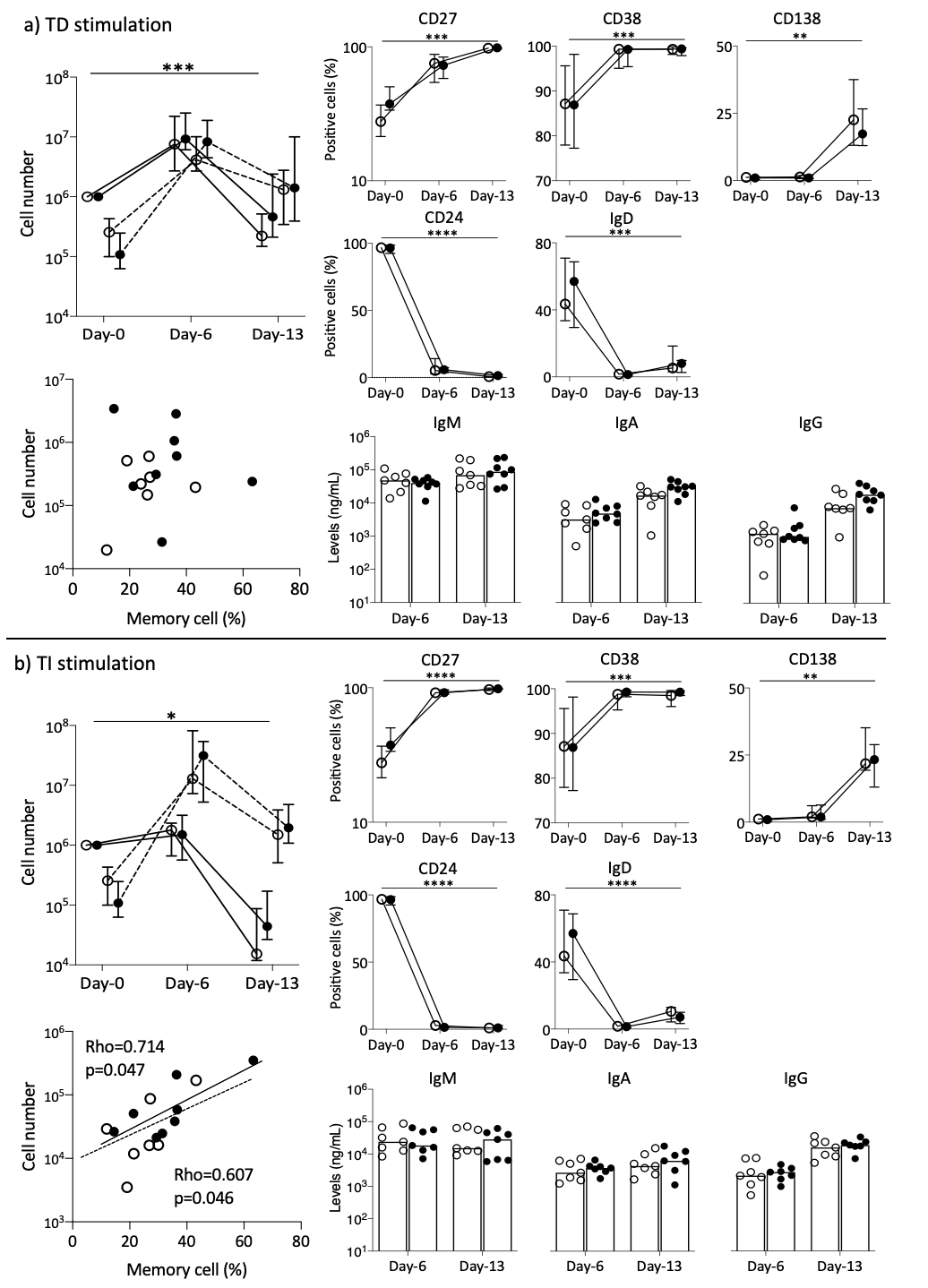


***Figure SUP-4.* Changes in B-cell differentiations between gender groups during a) TD and b) TI stimulation conditions.**

Top left :Number of live (plain line) and dead (dashed line) B-cells at different time points. Bottom left: Relationship between % memory cells in circulating B-cells and number of PC generated at day-13. *P*-value and rho were calculated using Pearson correlations.  Top right: Positivity (% of live cells) for each marker (median; IQR). Bottom right: Total IgM, IgA and IgG levels measured from supernatant collected at day-6 and 13. N=7 male and n=8 female (1 donor missing CD24, IgD and IgM/A/G in TI). *P*-value for the overall assays between groups were calculated by ANOVA (top bar). For individual comparison between 2 groups at a time point, MWU test results are given next to the group denoting difference between old and young donors. ○ male donors; ● female donors. *P*-values: # *p<0.100; * p=0.050; ** p<0.010; *** p<0.001; **** p=0.0001.*

**Figure SUP-5: Frequency of circulating B-cells subsets in the two age groups**. Younger (n=8) and older donor group (n=7); 4 subsets were quantify using classic flow cytometry: naïve (CD24^+^CD27^-^CD38^-^CD138^-^IgD^+^), memory (CD24^low^CD27^+^ CD38^low^CD138^-^IgD^-^), regulatory B-cells B-reg (CD24^high^CD27^-^CD38^high^CD138^-^IgD^+^) and early plasmablasts EPB (CD24^-^CD27^+^CD38^+^ CD138^low^IgD^-^). P-value calculated by MWU. # p=0.081; * p=0.019; N.S. not significant.

***Figure SUP-6:* B-cell changes in marker expression levels at** **day-13 of differentiation** **in younger and older subjects**. SPADE trees of cellular hierarchy for 8 markers expressed on B-cells, using the same subsets as in Figure 1. The only visible difference ( * ) is a larger number of cells in the EPB subset in the older donor in the TD assay while nothing appears noticeable in the TI assay.


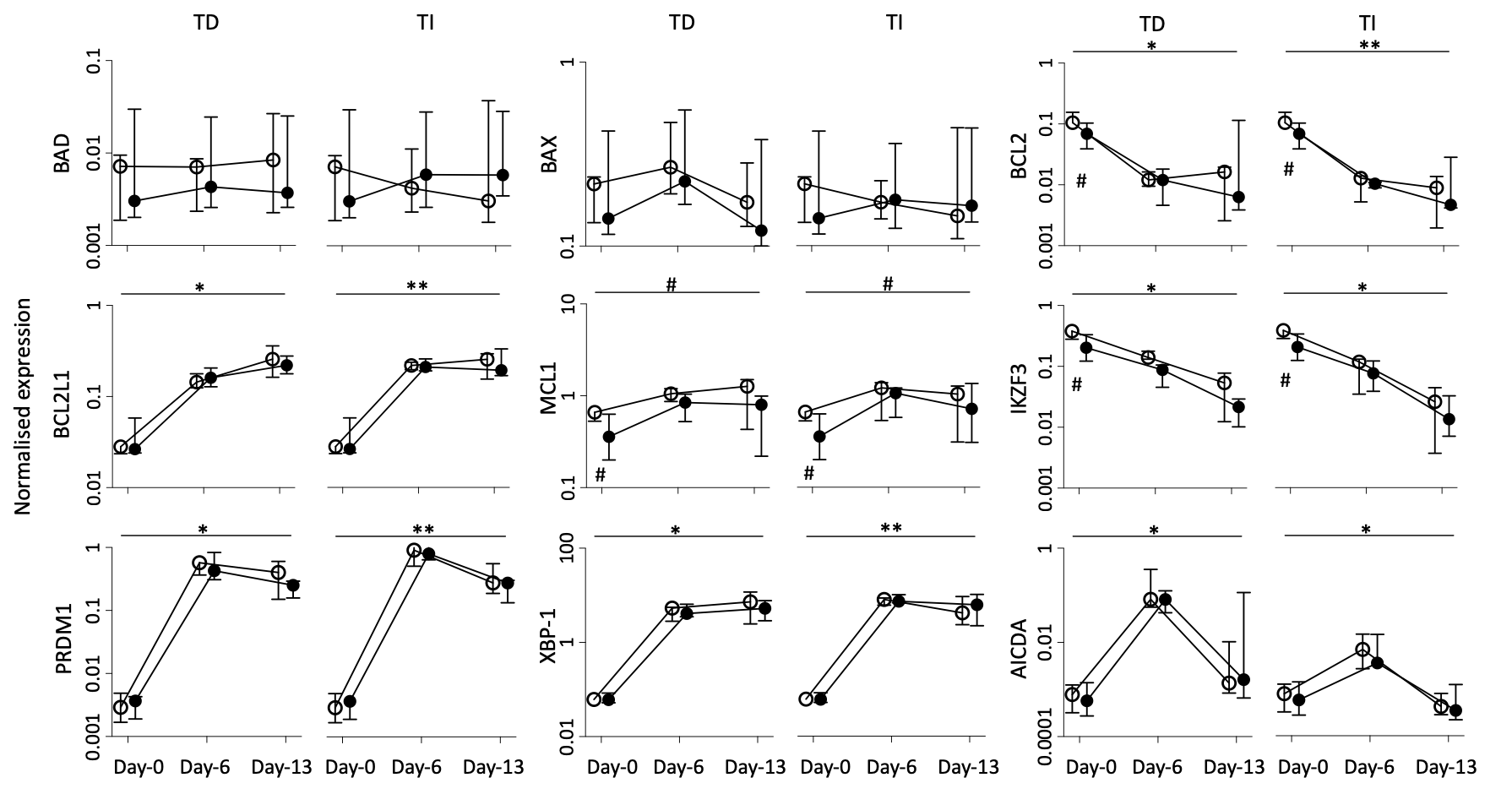


***Figure SUP-7.* Expression profile of gene associated with differentiation and survival/apoptosis.** Gene expression changes (median; IQR) during differentiation assays between gender groups at different time points, quantified by Taq-man qPCR (n=5 for each group). Overall changes between two groups were tested by ANOVA (top bar). Individual time point differences were tested by MWU. ○ male donors; ● female donors. *P*-values*: # p<0.100; * p<0.050; ** p<0.010.*
